# EHO-85, Novel Amorphous Antioxidant Hydrogel, Containing *Olea europaea* Leaf Extract—Rheological Properties, and Superiority over a Standard Hydrogel in Accelerating Early Wound Healing: A Randomized Controlled Trial

**DOI:** 10.3390/pharmaceutics15071925

**Published:** 2023-07-11

**Authors:** José Verdú-Soriano, Marisol de Cristino-Espinar, Silvia Luna-Morales, Caridad Dios-Guerra, Antonio Casado-Díaz, José Manuel Quesada-Gómez, Gabriel Dorado, Miriam Berenguer-Pérez, Susana Vílchez, Jordi Esquena, Leocadio Rodríguez-Mañas, José Luis Lázaro-Martínez

**Affiliations:** 1 Department of Community Nursing, Preventive Medicine, Public Health and History of Science, Faculty of Health Sciences, University of Alicante, 03690 Alicante, Spain; miriam.berenguer@ua.es; 2 Pharmacy Department, Reina Sofia University Hospital, 14004 Córdoba, Spain; ms.cristino.sspa@juntadeandalucia.es; 3 Maimonides Institute of Biomedical Research of Cordoba (IMIBIC), Reina Sofía University Hospital, University of Córdoba, 14004 Córdoba, Spain; silvialu67@gmail.com (S.L.-M.); cdiosguerra@gmail.com (C.D.-G.); md1qugoj@uco.es (J.M.Q.-G.); 4 Occidente Health Center, Córdoba and Guadalquivir Health Management Area, 14005 Córdoba, Spain; 5 Department of Nursing, Faculty of Medicine and Nursing, University of Cordoba, 14004 Córdoba, Spain; 6 Endocrinology and Nutrition Unit, Reina Sofia University Hospital, 14004 Cordoba, Spain; 7 Consortium for Biomedical Research in Frailty & Healthy Ageing (CIBERFES), Institute of Health Carlos III, 28029 Madrid, Spain; bb1dopeg@uco.es (G.D.); leocadio.rodriguez@salud.madrid.org (L.R.-M.); 8 Dep. Bioquímica y Biología Molecular, Campus Rabanales C6-1-E17, Campus de Excelencia Internacional Agroalimentario (ceiA3), Universidad de Córdoba, 14071 Córdoba, Spain; 9 Institute of Advanced Chemistry of Catalonia, Consejo Superior de Investigaciones Científicas (IQAC-CSIC), 08034 Barcelona, Spain; susana.vilchez@cid.csic.es (S.V.); jordi.esquena@iqac.csic.es (J.E.); 10 Networking Research Center on Bioengineering, Biomaterials and Nanomedicine (CIBER-BBN), Institute of Health Carlos III, 28029 Madrid, Spain; 11 Geriatric Research Group, Biomedical Research Foundation at Getafe University Hospital, 28905 Getafe, Spain; 12 Department of Geriatrics, University Hospital of Getafe, 28905 Getafe, Spain; 13 Diabetic Foot Unit, University Podiatry Clinic, Complutense University of Madrid, 28040 Madrid, Spain; diabetes@ucm.es

**Keywords:** amorphous hydrogel, EHO-85, diabetic foot ulcer, pressure ulcer, randomized active-controlled trial, venous leg ulcer

## Abstract

Many advanced wound healing dressings exist, but there is little high-quality evidence to support them. To determine the performance of a novel amorphous hydrogel (EHO-85) in relation to its application, we compared its rheological properties with those of other standard hydrogels (SH), and we assessed the induction of acceleration of the early stages of wound healing as a secondary objective of a prospective, multicenter, randomized, observer-blinded, controlled trial. The patients were recruited if they had pressure, venous, or diabetic foot ulcers and were treated with EHO-85 (*n* = 103) or VariHesive^®^ (SH) (*n* = 92), and their response was assessed by intention-to-treat as wound area reduction (WAR (%)) and healing rate (HR mm^2^/day) in the second and fourth weeks of treatment. Results: EHO-85 had the highest shear thinning and G′/G″ ratio, the lowest viscous modulus, G″, and relatively low cohesive energy; EHO-85 had a significantly superior effect over SH in WAR and HR, accelerating wound healing in the second and fourth weeks of application (*p*: 0.002). This superiority is likely based on its optimal moisturizing capacity and excellent pH-lowering and antioxidant properties. In addition, the distinct shear thinning of EHO-85 facilitates spreading by gentle hand pressure, making it easier to apply to wounds. These rheological properties contribute to its improved performance.

## 1. Introduction

The skin, a large and multifaceted organ, constitutes 15% of the body’s total weight, with fundamental vital functions, including protection against external physical, chemical, and biological aggressors. The skin is the first line of immune defense against infection and provides a shield against the harmful effects of solar ultraviolet radiation, which it uses to synthesize vitamin D3 and β-endorphins, which transmit painful and pleasurable stimuli, regulate body temperature, and maintain the body’s water and electrolyte balance [1].

Maintenance of the skin structure and function is critical to the health of the human body as a whole [2]. Therefore, as soon as any injury or break in the skin occurs, it sets in motion a sequence of overlapping events aimed at repairing the wound: (i) Hemostasis; (ii) inflammation; (iii) proliferation; and (iv) maturation/remodeling [3].

However, sometimes, wounds may not progress through the normal healing process and remain open for more than a month in a process of chronification [4]. Chronic wounds represent a silent epidemic, affecting a large part of the world’s population. In developed countries, it has been estimated that 1–2% of the population will experience a wound at some point in their lives [5]. That represents a major and growing threat to public health and economic systems. Indeed, acute and chronic wound problems have dramatically increased worldwide in recent years. Thus, they affect more than 305 million people, which, in addition to the physical and mental suffering, results in a financial burden of treatment of up to US $80 billion by 2024 [6]. Inducing faster wound healing, which prevents the process of chronification and prevents wound infections, is therefore a major challenge in modern medicine [7].

Hydrogel-based dressings are considered an ideal material for wound dressing, due to several advantages: (i) Three-dimensional structure, which facilitates their application [8]; (ii) facilitating debridement, without adherence to the underlying sensitive tissue; (iii) providing pain reduction through cooling; (iv) keeping wounds adequately moist; with (v) great capacity to incorporate many bioactive agents, enabling safe delivery of drugs, growth factors, peptides, stem cells, and/or other bioactive substances to the wound bed [9]. All of these factors facilitate the healing process, promoting adequate therapeutic compliance and generating an environment conducive to wound repair [10,11].

Recently it has been reported that the EHO-85 amorphous hydrogel, containing *Olea europaea* leaf extract, modulates the wound microenvironment. That is accomplished through its antioxidant effect, as well as the regulation of pH and wetting [12]. All that holistically cooperates with the natural physiological processes involved in wound healing [13,14]. Moreover, a prospective, parallel-group, randomized, investigator-blinded, and multicenter clinical trial has shown that EHO-85 improves wound healing rates compared to a standard amorphous hydrogel [14].

Therefore, the aim of the present study was to understand the performance of the EHO-85 amorphous hydrogel by evaluating its rheological properties vs. SH hydrogels, in relation to its applicability and ability to stimulate the acceleration of early wound healing from the initial days of the process.

## 2. Materials and Methods

### 2.1. Rheological Study

The rheological properties of the amorphous hydrogel EHO-85 were studied at 37 °C, and its properties were compared with those of four commonly used wound healing hydrogels: VariHesive (ConvaTec. Reading, UK), Purilon (Coloplast. Humlebaek, Denmark), Intrasite (Smith & Nephew. Watford, UK), and Nu-gel (Systagenix Wound Management. Gatwick, UK).

#### Viscosity and Oscillatory Rheological Tests

Two types of determinations, rotational and oscillatory, were performed using an AR-G2-controlled stress rheometer (TA Instruments, New Castle, DE, USA), equipped with plate–plate stainless-steel geometry with a 20 mm diameter and 1000 μm gap. Water evaporation was prevented with a solvent trap. All measurements were performed in duplicate, and the temperature was kept constant at 37 °C, adjusted with a Peltier control system.

Before each measurement, either rotational or oscillatory, gels were pre-sheared at 30 s^−1^ for 10 s and equilibrated for 5 min to obtain homogeneous conditions. The viscosity was obtained by rotational tests, increasing the shear rate from 0.001 s^−1^ to 500 s^−1^. Afterwards, strain sweeps were performed to determine the linear viscoelastic region in a strain range of 0.01% to 100%, at a frequency of 1 Hz. Finally, the viscoelastic properties (elastic and viscous modulus, G′ and G″, respectively) were measured by oscillatory determinations, applying a sinusoidal shear deformation to the samples and measuring the stress response, increasing the frequency from 0.01 to 10 Hz at a strain within the linear viscoelastic region.

The cohesive energy density, E_c_, was calculated from the value of G′ within the linear viscoelastic region and the critical strain (*γ_crit_*), which is the minimum strain that breaks the bonds of the structure. E_c_ (Equation (1)) is defined as the energy required to break the structure of viscoelastic fluids [15,16].

(1) $$E_{c}=\int_{0}^{\gamma_{crit}}\gamma_{o}G^{\prime }d\gamma =\frac{1}{2}\gamma_{crit}^{2}G^{\prime }$$

Data were analyzed using TRIOS software (TA Instruments, New Castle, DE, USA).

### 2.2. Clinical Trial: Patients and Procedures

#### 2.2.1. Design

This study is the secondary objective of a prospective, parallel-group, randomized, investigator-blinded, multicenter clinical trial, included in the trial to evaluate the superiority of the EHO-85 amorphous hydrogel in accelerating the initial phases of the wound healing process compared to a standard hydrogel widely used in the treatment of ulcers, which has been used for many years with efficacy and safety. The trial was approved by the Ethics Committee of Cordoba (Cordoba, Spain) and by the Spanish Agency for Medicines and Health Products (AEMPS) for testing a new medical device (PS/CR 623/17/EC). Additionally, it was conducted in accordance with ISO 14155 “Clinical investigation of medical devices for human subjects: good clinical practice” and the Declaration of Helsinki. All participants gave written informed consent. The full details of the pivotal clinical trial are described in a previous publication [14].

#### 2.2.2. Patient Inclusion and Exclusion Criteria

Patients of both sexes (≥18 years old) were recruited if they had a diagnosis of venous leg ulcers (VLU), pressure ulcers (PU), category II (partial thickness), or III (full thickness with skin loss). This was carried out according to the European Pressure Ulcer Advisory Panel (EPUAP) [17], or diabetic foot ulcers (DFU), grade I or II according to the Wagner scale [18] of neuropathic origin excluding ischemic conditions. All ulcers had to have an evolution of between 1 and 36 months and an area between 1 cm^2^ and 1.99 cm^2^. If there was more than one ulcer, the one that best met the selection criteria (target ulcer) was selected.

Exclusion criteria were severe renal or hepatic insufficiency, connective tissue disease, systemic or local wound infection, pregnancy and lactation, HbA1c > 9.5%, or serum albumin < 2.5 g/dL. Patients were also excluded if they were receiving treatment with systemic corticosteroids, immunosuppressants, tumor necrosis factor (TNF) inhibitors (for the treatment of rheumatoid arthritis), or PPAR-gamma agonists (for the treatment of diabetes). Patients with VLU and DFU had to have a preserved posterior tibial and/or a pedal pulse and the ankle-brachial pressure Index (ABPI) had to be ≥0.8. Other specific exclusion criteria were defined for each type of ulcer. The baseline characteristics of patients and the descriptions of ulcers and prior treatments are shown as Supporting Information (Tables S1 and S2, respectively) obtained from the pivotal clinical trial [14]. In the case that wound debridement or infection control were required, the start of treatment was delayed from 24 to 72 h after the completion of debridement.

#### 2.2.3. Product under Investigation, Comparator, and Treatment Description 

EHO-85 is a class IIb medical device in the form of an amorphous hydrogel, composed of (i) purified water; (ii) Carbopol 980^®^, an easily and rapidly dispersible cross-linked acrylic acid polymer [19], which helps to provide an insulating and protective barrier to the ulcer bed; (iii) *Olea europaea* leaf extract (OELE), the most important and distinctive of its components, included for its ability to regulate free radicals in the ulcer bed [13]; (iv) triethanolamine (TEA) used as an agent for polymer gelation and gel network formation [19]; (v) Geogard Ultra^®^ (gluconolactone, sodium benzoate, and calcium gluconate) to prevent microbiological contamination; (vi) disodium salt of ethylene diamine-tetraacetic acid (Na_2_-EDTA for its antimicrobial and antibiofilm properties [20]; (vii) Glycerin [21]; and (viii) Fucocert^®^ (L-fucose, D-galactose, and galacturonic acid) [22], included as moisturizing and self-emulsifying agents, key in repair and elasticity, which help to create a protective film on the wound. The combination of these components, together with the formulation’s ability to reduce the alkalinity of the ulcer bed through its slightly acidic pH (5.0–5.5), gives EHO-85 amorphous gel the ability to modulate the wound environment, promoting and accelerating the healing process [13].

Regarding the organoleptic characteristics of the EHO-85 amorphous hydrogel, its appearance was that of a homogeneous and translucent gel, odorless and discreetly pale yellow in color. Among its physical and chemical characteristics are (i) slightly acidic pH, (ii) a density of 1.05 to 1.10 g/mL at 20 °C, and (iii) viscosity (speed 5v5 rpm, 20 °C) of 35,000–50,000 centipoises. According to the European Standard BS EN 13726-1, in relation to absorbency properties, EHO-85 has a significant wetting capacity (type D) and is partially dispersible [13].

The positive control product was another amorphous hydrogel (VariHesive^®^, ConvaTec, Barcelona, Spain), a reference product that is marketed in other countries under other brand names such as Duoderm Hydroactive Hydrogel^®^, Duoderm Hydroactive Sterile Gel^®^, Duoderm Gel^®^, or GranuGel^®^. It is composed of sodium salt of carboxymethylcellulose, pectin, propylene glycol, and water.

The care protocol was identical in both groups. A thin layer (2 to 3 mm) was applied to the wound, and up to 5 mm in the case of cavitated ulcers. Both products were applied 3 days a week and, whenever possible, on alternate days. The target wound was cleaned using sterile saline. A silicone foam dressing (Mepilex^®^, Molnlycke, Gothenburg, Sweden) was then applied. No other general or local treatment was allowed. Compression therapy was mandatory among patients with VLU. An elastic compression bandage (Indacrep^®^, Inda, Barcelona, Spain) was applied over the secondary dressing [23]. The dressing was changed every 24 or 48 h (depending on the needs of each patient). Similarly, patients with PUs had to comply with a pre-specified standardized repositioning regimen. Patients with DFU used foam cushioning in combination with appropriate footwear. In the case of ulcer infection during the study period, the frequency of cleaning and secondary dressing changes was intensified. In addition, if necessary, a nanocrystalline silver dressing (Acticoat^®^/Argencoat^®^; Smith&Nephew) was applied to the ulcer until remission of the infection. The Clinical Investigation Plan did not foresee the discontinuation of the treatment products. However, the final decision was at the discretion of the investigator, who could discontinue treatment for this reason if justified.

#### 2.2.4. Randomization and Stratification

Stratified randomization was performed using REDCap^®^ version 7.06 (Research Electronic Data Capture System, Vanderbilt University, Nashville, TN, USA) research electronic data capture software (REDCap^®^), programmed for this purpose by the Innovation Department of the Maimonides Institute for Biomedical Research of Cordoba. The stratification, at the first level, considered the etiology of the ulcer (VLU, PU, or DFU), and then the duration of the ulcer using a cut-off point of six months and the area of the ulcer (cut-off point of 10 cm^2^).

#### 2.2.5. Procedures

After obtaining written informed consent from patients or legal representatives to participate in the trial, demographic parameters, patient medication, and medical, surgical, and ulcer history were documented at the screening visit. Patients eligible for the study were recruited and randomly assigned to each of the two groups (EHO-85 or VariHesive^®^). Ulcers were assessed by the research nurse every two weeks until week eight. After cleansing the wound with sterile saline solution, at each visit, two wound photographs of at least eight megapixels were taken and sent to an experienced, software-trained principal investigator (who had not participated in the trial regarding applying treatments and was unaware of the type of dressing applied). Wound images displayed a standardized label including date, patient code, and a millimeter ruler used to enable digital wound planimetry and granulation tissue area measurements (Pictzar Pro^®^ version 7.5.1; Advanced Planimetric Services, Elmwood Park, NJ, USA [24]).

#### 2.2.6. Effectiveness Assessment Criteria

The main outcomes of the study designed to confirm whether EHO-85 gel promotes and/or accelerates the wound healing process included (i) absolute wound area reduction (WAR), calculated as (A_t_ − A_0_), where A_t_ is the last measurement of wound area and A_0_ is the initial wound area, expressed in mm^2^; (ii) relative WAR calculated as [(A_t_ − A_0_)/A_0_ ] × 100 and expressed as a percentage (%); and (iii) daily wound healing rate [(A_t_ − A_0_)/t, expressed in mm^2^ per day of treatment, where t is the time after starting treatment.

#### 2.2.7. Data Collection and Database and Statistical Analysis

An electronic data collection form based on REDCap^®^ was used. Data were anonymized and measures were taken to ensure the confidentiality of patient data. 

Statistical analyses were performed at the IMIBIC Innovation Department, using R 4.0.3 software (R Foundation for Statistical Computing, Vienna, Austria). All analyses were performed on an “intention-to-treat” (ITT) population.

A statistical description of all variables involved in the study was made, including measures of the central tendency (mean, median, and mode) and dispersion (range and standard deviation). Median differences are given with a 95% confidence interval (CI). Ordinal and nominal variables are presented by the number of patients involved and the percentage. The initial comparability of the two groups was verified by adapted tests (Student’s *t*-test, Mann–Whitney non-parametric test, and chi-square), depending on the distribution and nature of the variables. Since the Shapiro–Wilk test concluded that the quantitative variables were not consistent with normality, the non-parametric Wilcoxon–Mann–Whitney test was used to assess the homogeneity of the baseline variables with respect to the group (intervention and control).

## 3. Results

### 3.1. Viscosity and Oscillatory Rheological Tests

**Figure 1:**
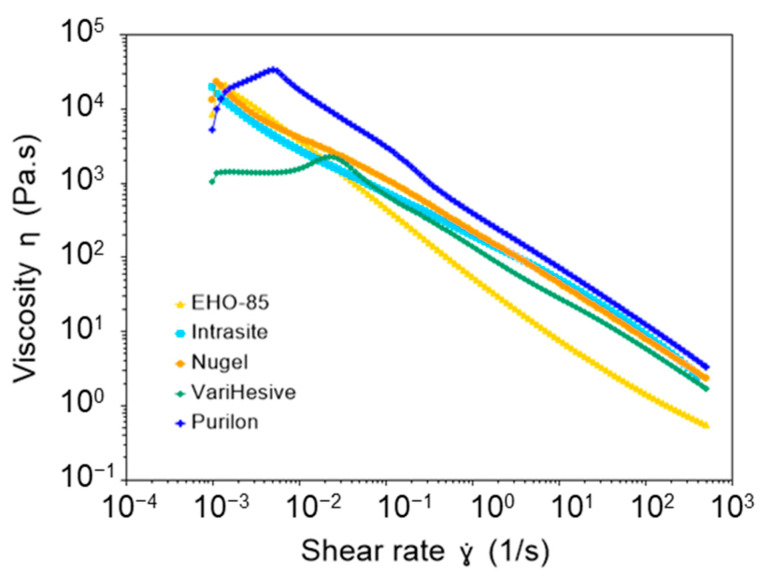
Comparison of the viscosity of EHO-85, vs. Intrasite, Nu-gel VariHesive, and Purilon samples.

The rotational measurements performed to determine viscosity showed that all samples were non-Newtonian (viscosity was not independent of the shear rate). In addition, they showed pseudoplastic behavior since viscosity decreased with the shear rate (Figure 1 and Table 1).

VariHesive showed the lowest viscosity at rest (low shear rate). EHO-85 had similar behavior to Nu-gel and Intrasite at a low shear rate, showing viscosities at 0.01 s^−1^ of 3512, 2702, and 3877 Pa·s, respectively. However, EHO-85 viscosity considerably decreased when increasing shear (shear thinning behavior), in comparison to other samples. EHO-85 had the lowest viscosity at a high shear rate, above 0.1 s^−1^ (Figure 1 and Table 1). Therefore, although shear thinning occurred in all samples, EHO-85 exhibited the most pronounced behavior.

Although all studied samples were viscoelastic hydrogels with shear thinning behavior, EHO-85 was the hydrogel with the most pronounced shear thinning. This demonstrates a higher spreadability compared to other samples. EHO-85 also showed the highest G′/G″ ratio, with the lowest viscous modulus, G″. In addition, the elastic modulus, G′, was quite independent of the frequency. It is most likely that these properties facilitate spreading on the skin, by applying gentle hand pressure.

Two replicates of each determination were carried out. The experimental error is quite low since background noise is not observed in the measurements. Moreover, the two replicates provided very similar results in all five hydrogel samples (Figure S1, in Supporting Information). Consequently, it was concluded that the results were highly reproducible. Viscoelastic determinations by oscillatory assays also provided good reproducibility among the two replicates. 

The visual aspect of samples in the absence of shear is also shown as Supporting Information (Figure S2). In these conditions, Intrasite and Nu-gel were stiff gels, whereas VariHesive appeared to be the most fluid hydrogel. EHO-85 and Purilon appeared to show intermediate fluidity. These visual observations agree with the starting points of the flow curves at the lowest shear rate of 0.001 s^−1^ (Table 1), since more viscous samples showed less fluidity at rest. 

### 3.2. Viscoelastic Properties

Viscoelastic behaviors were evaluated by oscillatory measurements. In strain sweep assays, samples were deformed from small to high amplitudes to study their elastic modulus, G′ (energy storage), and viscous modulus G″ (energy loss) across the strain range (Supporting information, Figure S3). Each plot was divided into two sections: The region of low strain, where both moduli remained constant; and the region of high strain, where moduli decreased. Linear viscoelastic regions ended at γcrit, where G′ started to decrease. All samples showed typical viscoelastic behavior with high values of elastic moduli, confirming that they behaved as gels. 

Strain assays were performed to find viscoelastic regions, where G′ was independent of strain. Afterwards, frequency assays were performed by applying strain within this region. EHO-85, Purilon, Intrasite, and Nu-gel samples exhibited an elastic modulus, G′, higher than their viscous modulus, G″ (Supporting Information Figure S4). These results demonstrated that their behavior was predominantly elastic. VariHesive behaved differently, with elastic and viscous moduli of very similar values. EHO-85 had the highest G′/G″ ratio, with the G′ elastic modulus similar to other tested gels but with a much lower G″ viscous modulus.

**Figure 2:**
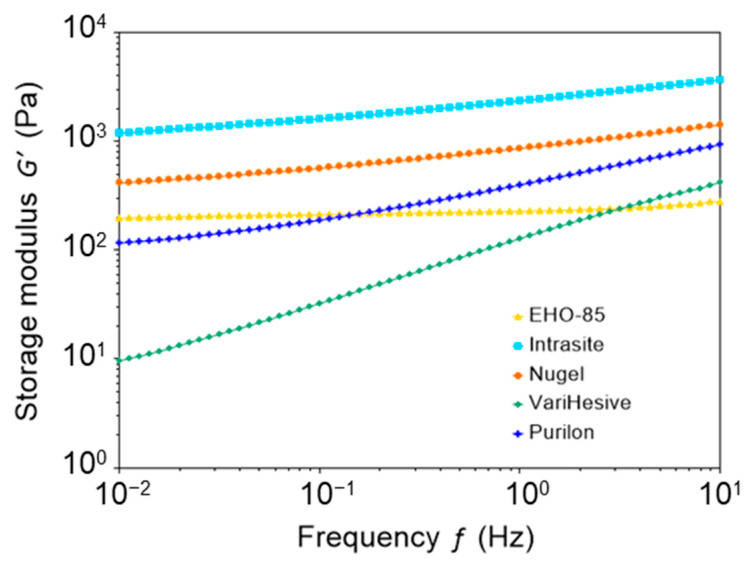
Elastic modulus (G′) of EHO-85, Intrasite, Nu-gel, VariHesive, and Purilon samples, as a function of oscillation frequency at constant strain.

Figure 2 shows the elastic modulus (G′), as a function of oscillation frequency, for all tested hydrogels. Remarkably, the elastic modulus of EHO-85 was almost constant from 0.01 Hz to 10 Hz, which indicates the presence of a more stable structure, as compared to other hydrogel samples. In contrast, other gels showed a frequency dependence of the elastic modulus. The values of G′ at 1 Hz are shown in Table 2.

The cohesive energy density, Ec, is related to the strength of the structure that maintains the gel texture. Ec was calculated applying Equation (1), measuring *γ_crit_* as the value of the strain that starts decreasing G′ (using the arbitrary criteria that G′ decreases when it is 5% below its average value at the linear viscoelastic region). The values of Ec were 0.067, 0.023, 1.12, 12.3, and 9.73 J/m^3^ for EHO-85, Intrasite, Nu-gel, VariHesive, and Purilon, respectively (Table 2). Therefore, EHO-85 had a rather small cohesive energy, 0.067 J/m^3^, indicating that the interactions that sustain the structure are rather weak and might be reversible.

### 3.3. Clinical Trial Results

**Figure 3:**
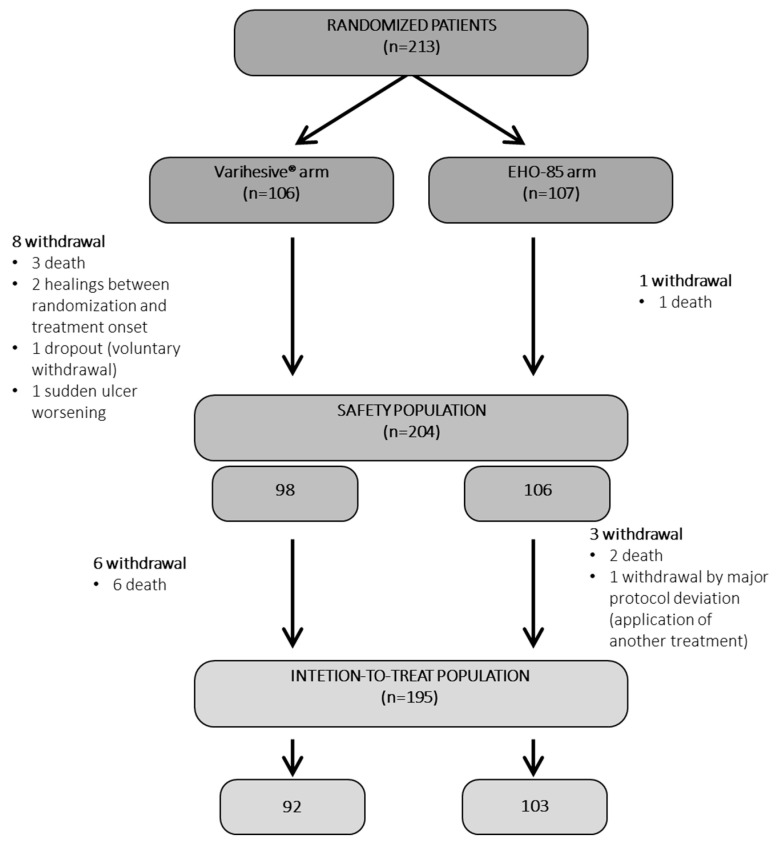
Patient flow diagram. All socio-demographic data, ulcer characteristics, and previous local treatments were well balanced between the two groups at baseline (Supplementary information Tables S1 and S2). The same applied to PU, VLU, and DFU when their baseline data were separately analyzed.

A total of 213 patients from 23 health centers were included in the pivotal RCT. They were randomized to receive either the EHO-85 amorphous hydrogel (*n* = 107) or the positive control VariHesive^®^ (*n* = 106). Eighteen could not be included in the ITT analyses: Four in the EHO-85 group and fourteen in the VariHesive group (Figure 3). Therefore, 195 patients (92%) comprised the ITT studied populations: EHO-85 amorphous hydrogel (*n* = 92) vs. VariHesive control (*n* = 103).

**Figure 4:**
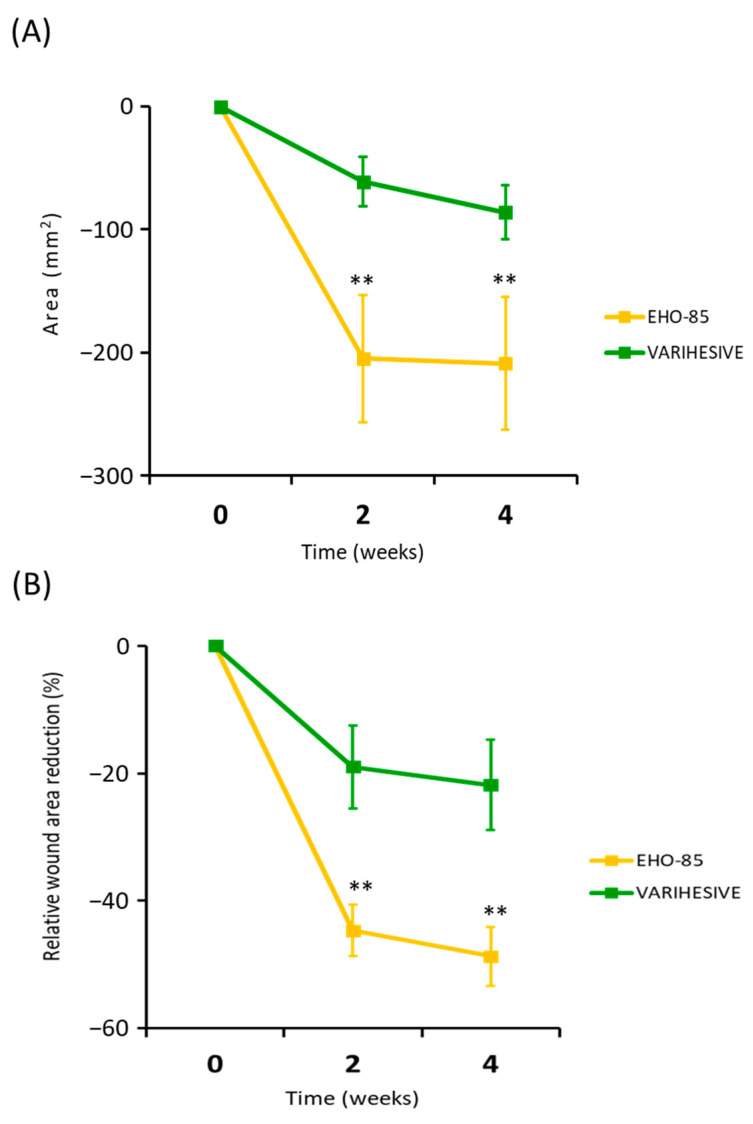
Evolution of the reduction of the ulcer area in both treatment groups during the 28 days of follow-up. (**A**) Absolute WAR. (**B**) Relative WAR evolution ITT analysis. Results are expressed as mean ± SD. ** *p* < 0.01 for the comparison of EHO-85 vs. VariHesive^®^ after the second and fourth weeks of treatment. Significant and continued improvements in the WAR from the first days of application show EHO-85 acts as a trigger for the healing process.

Responses to treatments in relation to reductions in ulcer areas in the first weeks of administration as an evaluator of their effectiveness triggering the acceleration of healing processes are shown in Figure 4.

Results showed that most of the EHO-85 amorphous hydrogel effects, promoting and accelerating wound healing, were concentrated in the first few days of application. Thus, the average ulcer closure was 26% higher among patients after only 14 days of treatment (–44.8 ± 38.8% vs. –18.7 ± 59.2%; *p* = 0.002). This effect tended to increase over the next weeks, albeit at a slower rate (Figure 4).

**Figure 5:**
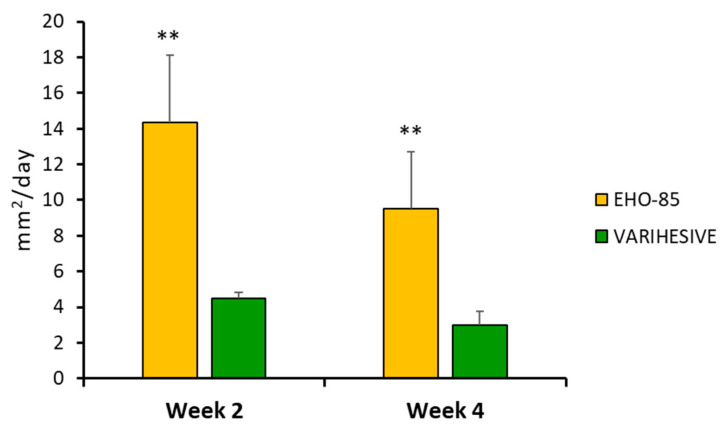
Daily ulcer reduction in the two treatment groups, in weeks 2 and 4, expressed in mm^2^. ITT population. Results are shown as mean ± Standard Error of the Mean (SEM). ** *p* < 0.01 for EHO-85 vs. VariHesive after the second and fourth weeks of treatment.

This superiority is also shown in the average daily closing speed (in mm^2^) between the two groups. Thus, after just six product applications, the closure rate in the EHO-85 hydrogel treatment group was three times higher (14.4 ± 38.0 vs. 4.5 ± 3.0 mm^2^/day; *p* < 0.01) (Figure 5).

## 4. Discussion

The viscosity study shows that EHO-85 has a more pronounced shear thinning. The high viscosity at low shear rates confirms that this amorphous hydrogel does not flow at rest, allowing controlled dosing when initially removed from the container (as also observed by manual handling). However, the lower viscosity at higher shear rates facilitates easy extension of the sample by applying soft manual pressure [25]. Shear thinning occurred in all evaluated samples, but EHO-85 exhibited a lower viscosity, which translates into a higher spreadability. EHO-85 gel is primarily formulated with an anionic acrylic polymer (carbopol 980^®^) with cationic ethanolamine as a counterion, which could result in low viscosity at a high shear rate [26,27].

The ease with which EHO-85 can be applied to wounds (spreadability) is relevant for the performance of the product. Manual spreading of the EHO-85 on the wound reduces the thickness of the sample, increasing its surface area [25]. Consequently, the shear rate (defined as the velocity of the liquid divided by the thickness of the liquid film) is high during sample spreading (typically in the order of 10^2^ s^−1^). The EHO-85 amorphous hydrogel showed the lowest viscosity at high shear rates, resulting in a better ability to pull out of the tube, and spread over the wound by applying gentle manual pressure.

These characteristics make the EHO-85 amorphous hydrogel easier to apply to wounds, as compared to other conventional amorphous hydrogels, enabling better application and thus facilitating better compliance in wound treatments.

This multicenter, randomized, controlled, and blinded clinical trial has demonstrated that patients treated with EHO-85 amorphous hydrogel dressing showed a significant acceleration of the wound healing process. That was observed from the first days of treatment, as compared to those treated with a standard hydrogel. Both groups received good standard care, as recommended in the guidelines. Rapid wound healing is a therapeutic challenge, because prolonged or chronic wound healing causes significant biological, psychological, social, and economic burdens, for patients, caregivers, and public health systems in general [28,29].

So far, according to the most recent guidelines and systematic reviews, the evidence supporting the adoption of a particular intervention in the management of ulcers/skin lesions is sparse and inconsistent [30]. In fact, until now, it was unclear whether different hydrogels have different short-term effects. Most trials in this field are very small and poorly reported, so there was a great need for strong evidence from studies using high-quality methods such as the one we present [30].

The results of the present clinical trial are of clinical relevance that gives it particular strength and importance in relation to the state of the art by confirming that modulation of the lesion microenvironment, in conjunction with the best standards of treatment, depending on the etiology of the ulcer, achieves early promotion/acceleration of the healing process of skin ulcers [31,32]. 

This aspect is of particular importance in the field of amorphous hydrogels, in which EHO-85 is included. Previous systematic reviews to date have not provided conclusive evidence to conclude that there are differences in effectiveness between different hydrogels, or between the use of hydrogels and other topical dressings/products, in relation to the healing of pressure ulcers [30], diabetic foot ulcers, or venous ulcers [33].

The ability of EHO-85 amorphous hydrogel to accelerate healing can be attributed to the modification caused by its application to the ulcer microenvironment [12,13], typically characterized by alkaline pH and an excess of reactive oxygen species (ROS) [13,34,35,36]. EHO-85 amorphous hydrogel combines moisturizing and barrier function properties, with a novel ability to down-regulate ROS and pH, modulating the ulcer microenvironment in a favorable holistic manner [37,38]. Generating a warm and moist environment promotes wound healing [39], so maintaining moist conditions is a crucial challenge for wound management [40]. Amorphous hydrogels, such as EHO-85, have shown excellent biochemical and mechanical properties that provide a three-dimensional scaffold that works as a supporting structure in the wound bed. In addition, it exhibits softness and flexibility (shear thinning), as well as biocompatibility. The rheology results clearly demonstrate that EHO-85 possesses better spreadability than other hydrogel samples. This allows the application of the product by soft hand pressure on wounds, facilitating homogenous coverage of the wound area with little applied force. Moreover, it has the ability to donate and/or absorb fluids, without dissolving them. That enables the generation of a moist environment, which is beneficial for wound healing [41]. In addition, such material is comfortable and easy to change. All that alleviates the pain of wounded tissue by generating a barrier of thermal insulation and mechanical protection. Furthermore, it adapts to the wound, while, as a highly permeable platform, it allows the diffusion of nutrients, metabolites, and water-soluble molecules, the exchange of simple gases, and the infiltration of cells. All these factors contribute to and explain the promotion and intensifying healing effects, as observed even from the first days of application. Because of all these characteristics, amorphous hydrogels are currently considered a reference among wound treatment dressings. They can be used in all types of ulcers (venous, pressure, diabetic, surgical wounds, burns, etc.), at any stage of the healing process [42]. For this purpose, the EHO-85 hydrogel was designed with the appropriate proportions of glycerin and Fucocert, which are highly moisturizing components, providing significant fluid donation capacity [14].

Another key effect of EHO-85 amorphous hydrogel in improving wound healing is the control and reduction of wound pH [43]. The administration of EHO-85 is able to induce an acidic environment in the wound bed from the first application, maintaining it over time. Interestingly, it is known that acidic pH inhibits pathogen growth. Such induction of a moderately acidic wound environment supports the natural barrier function, helping to counteract potential microbial colonization [44,45]. Acidification also contributes to enhancing various reparative processes. This is accomplished by promoting angiogenesis and improving the physiological activity of macrophages and fibroblasts [46] and favorably modifying the quantity and quality of matrix metalloproteinases. Interestingly, a reduction in wound pH may also directly influence the release of tissue oxygen into the wound [47]. Therefore, acidification of the pH of the ulcer medium, such as that induced by EHO-85, is an important contributor to healing [48]. This has been supported by several studies and clinical trials, showing that skin ulcers with a highly alkaline pH had lower healing rates compared to lesions whose pH was closer to neutral or acidic pH [49,50].

In the microenvironment of wounds that heal slowly, tend to become chronic, or have become chronic, there is an uncontrolled high level of reactive oxygen species (ROS), resulting in the deterioration of cell membranes. ROS also causes the degradation of peptides/proteins, lipids, and deoxyribonucleic acids (DNA and RNA). This keeps the wound in a state of chronic uncontrolled inflammation, which may lead to a delay or cessation of ulcerated tissue repair, cell proliferation, and angiogenesis [51].

The modulation of ROS to avoid excessive and sustained increases in oxidative stress over time is, therefore, a relevant objective during wound healing, significantly contributing to its acceleration [36,51]. In fact, some authors have explicitly proposed the urgency of developing hydrogel dressings with antioxidant properties. In this regard, the powerful antioxidant capacity of flavonoids, phenols, and oleuropeosides, extracted from OELE and included in EHO-85, has been extensively evaluated [12,13,52]. Oleuropein is the most abundant phenolic compound in OELE, followed by hydroxytyrosol, oleuropein, aglycone, and tyrosol, with the antioxidant activity of them being synergistic [53]. Indeed, it has been shown in several experimental animal models that the application of oleuropein or OELE to wounds significantly accelerates the healing of skin ulcers [54,55,56]. To a large extent, this ability was mediated by their antioxidant properties [54].

## 5. Conclusions

The available evidence for the management of skin wounds is limited and inconsistent, particularly in the knowledge of the use of hydrogel dressings. No evidence is available on the effect of hydrogels on the important early days of wound healing. We have reported preclinical and clinical evidence with EHO-85 hydrogel [12,13] and the overall results of a clinical trial in which EHO-85 was shown to improve wound healing rates compared to a standard amorphous hydrogel widely used worldwide [14].

The added value of this study is that the present clinical investigation could constitute a relevant milestone for evidence-based decision making in the treatment of cutaneous wounds in terms of accelerating early wound healing, as it is a controlled, prospective, randomized, multicenter, controlled clinical trial with a blinded assessor (highest level of evidence), which demonstrates the efficacy and superiority of the multifunctional amorphous hydrogel EHO-85 over another standard hydrogel commonly used in wound healing. In both treatment groups, the usual treatment standards were applied, including the same secondary dressing. All of these factors give the present research great strength as high-quality evidence. Therefore, the amorphous hydrogel EHO-85 can be proposed with a strong recommendation grade (A) in evidence-based clinical practice guidelines [57]. Furthermore, rheology results have demonstrated that EHO-85 is more spreadable than other tested hydrogels, showing a more intense shear thinning behavior. This product can be applied to wounds with soft hand pressure, facilitating the deposition on wounds with gentle manipulation. 

The ease of application of the amorphous hydrogel EHO-85, due to its rheological properties, and the acceleration it induces in early wound healing may contribute to better wound care compliance by patients and caregivers.

## Figures and Tables

**Table 1:** Viscosity of gel samples at different shear rates. Two replicates were performed, and the results are shown as mean ± SD.

	Shear Rate (s^−1^)				
Sample	0.01	0.1	1	10	100
EHO-85	3512 ± 449	428.7 ± 46.3	51.6 ± 4.2	7.5 ± 0.4	1.4 ± 0.1
Intrasite	2702 ± 125	754.4 ± 13.7	209.6 ± 15.9	50.8 ± 1.4	9.1 ± 0.1
Nu-gel	3877 ± 287	1063 ± 89.7	216.5 ± 12.4	43.6 ± 1.4	7.8 ± 0.2
VariHesive	1393 ± 213	634.9 ± 73.6	130.0 ± 13.3	26.8 ± 1.8	5.8 ± 0.2
Purilon	16,158 ± 1811	2901 ± 102	360.2 ± 13.5	67.8 ± 2.5	11.6 ± 0.3

**Table 2:** Elastic modulus (G′) at 1 Hz frequency and cohesive energy density (Ec). Two replicates were performed, and the results are shown as mean ± SD.

Sample	Elastic Modulus (G′) (Pa)	Cohesive Energy (E_c_) (J/m^3^)
EHO-85	227.1 ± 1.5	0.067 ± 0.001
Intrasite	2402 ± 64	0.023 ± 0.012
Nu-gel	867.0 ± 9.8	1.12 ± 0.02
VariHesive	121.5 ± 8.6	12.3 ± 1.5
Purilon	399.9 ± 4.1	9.73 ± 1.18

## Data Availability

Data supporting the findings of this study are available from the corresponding author upon request.

## Supplementary Materials

The following supporting information can be downloaded at: https://www.mdpi.com/article/10.3390/pharmaceutics15071925/s1, Figure S1. Flow curves, showing the viscosity as a function of shear rate (two replicates), for the five hydrogel samples (EHO-85, Intrasite, Nu-gel, VariHesive and Purilon); Figure S2. Visual aspect of the different gel samples (EHO-85, Purilon, VariHesive, Intrasite and Nu-gel); Figure S3. Strain sweep of gel samples, showing the storage (elastic) modulus G’ and the loss (viscous) modulus G” as a function of the strain; Figure S4. Frequency sweep of gel samples, showing the storage (elastic) G’ and the loss (viscous) modulus G” as a function of frequency; Table S1. Baseline characteristics of patients; Table S2. Description of Ulcers and Prior Treatments; Table S3. List of collaborating nurses (sub-investigators) by centers.
